# Grief and the inconsolation of philosophy

**DOI:** 10.1017/S0031819123000049

**Published:** 2023-04-03

**Authors:** Dominic JC Wilkinson

**Affiliations:** 1 Oxford Uehiro Centre for Practical Ethics, Faculty of Philosophy, University of Oxford, UK; 2 John Radcliffe Hospital, Oxford, UK; 3 Murdoch Children’s Research Institute, Melbourne, Australia; 4 Centre for Biomedical Ethics, National University of Singapore Yong Loo Lin School of Medicine, Singapore

## Abstract

Can metaphysics yield the consolations of philosophy? One possibility, defended by Derek Parfit, is that reflection on the nature of identity and time could diminish both fear of death and grief.

In this paper, I assess the prospect of such consolation, focussing especially on attempts to console a grieving third party. A shift to a reductionist view of personal identity might mean that death is less threatening. However, there is some evidence to suggest that such a shift does not necessarily translate into less death anxiety. Moreover, applied to grief at loss of another, such a perspective may be misdirected. A temporally neutral perspective offers a theoretically powerful way of reducing the sense of loss at being separated in time from a loved one. However, it is unclear whether it is psychologically possible to achieve. Even if it were possible, it may not diminish the pain of separation.

I identify a serious challenge to philosophical consolation for grief. The greater the consolation that is offered, the greater the risk of losing important attachments and the less it may be psychologically accessible.

## Introduction

‘I am very sorry to learn that Ray died a couple of weeks ago. When someone I loved died I found it helpful to remind myself that this person was not less real because she wasn’t real now, just as people in New Zealand aren’t less real because they aren’t real here.’ —Derek Parfit^1^

Philosophy, by providing reasons or arguments that would ease our fears or worries, might offer us consolation. Since at least the ancient Greeks, philosophers have suggested that rational reflection about the nature and meaning of death could alleviate anxiety and fear at the prospect of dying. For example, Epicurus wrote: ‘a right understanding that death is nothing to us makes the mortality of life enjoyable, not by adding to life an unlimited time, but by taking away the yearning after immortality’ (Epicurus).

If they are right, a different form of consolation might be offered − philosophy might help to ease our pain at the death of a loved family member or friend. Seneca claimed that philosophy might be used to permanently still such feelings (Seneca et al., 2016, p. 183).

Finally, and undoubtedly the hardest challenge: if it can assuage our own grief, perhaps we may be able to draw on philosophy to help console others who are overcome by the loss of someone close to them? It is perhaps a mark of the difficulty of this, that consolation of third parties who are grieving has received relatively little philosophical attention.^2^

The idea of a philosophical consolation for mortality matters because these are common concerns. They are profound and often intensely distressing, both in prospect and (for bereavement) in experience.

Religious traditions often provide attractive and accessible responses to these challenges. For example, many religions offer the possibility of continued existence after death of the physical body as a balm for the fear of dying. In response to the loss of a family member or friend, religions sometimes offer the possibility of communicating with the deceased, or console with the claim that it will be possible to see the loved one again in the afterlife. Can philosophy offer anything that compares?

Most secular philosophies do not and cannot offer the option of surviving beyond bodily death.^3^ Rather than denying that we will die, philosophy must offer ways to come to terms with the reality of our death. For grief, philosophy must find a way to diminish the powerful sense of loss felt when someone loved is no longer there. It cannot pretend that we will be reunited in the world beyond.

The contemporary philosopher, Derek Parfit, particularly in his book ‘Reasons and Persons’, proposed some radical changes in the way that we should think about both identity and time. He articulated a view of personal identity that diminishes a strong sense of self. Furthermore, he suggested that we should try to reduce our bias towards the future, and adopt a more ‘timeless’ attitude to the past. He suggested that these changes in perspective had reduced his own fear of dying and helped him to deal with the death of others. As the quote at the start of this paper indicates, he also tried, on occasion, to help others with these insights.

In this paper, I will draw on Parfit’s philosophy to examine the potential for philosophical consolation in the face of death and bereavement. I first outline the relevant elements of his work and how they could be applied to the problem of mortality. I will then explore some of the philosophical and empirical challenges to Parfit’s consolation. I argue that impersonal and impartial approaches to philosophy might succeed in reducing anxiety at one’s own death (though existing empirical evidence does not support this). However, they potentially miss the inextricably personal, partial and temporally rooted nature of grief. Further, they can potentially reduce sadness at the death of another only at the cost of reducing our attachment to those we love. That cost, if it were achievable, might be too great. Finally, the more powerful the claimed consolation, the more challenging it appears to be to achieve the required change in mindset. This is one reason why such approaches are potentially impotent in the face of another person’s grief. Although I focus on Parfit, these challenges are potentially shared with other rational philosophical approaches.^4^

## Parfitian consolation

‘When I believed [that personal identity is what matters], I seemed imprisoned in myself. My life seemed like a glass tunnel, through which I was moving faster every year, and at the end of which there was darkness. When I changed my view, the walls of my glass tunnel disappeared. I now live in the open air. There is still a difference between my life and the lives of other people. But the difference is less. Other people are closer. I am less concerned about the rest of my own life, and more concerned about the lives of others’ (Parfit, 1984, p. 281).

In his seminal work, Reasons and Persons, Parfit set out to interrogate and challenge some conventional ways of thinking about our nature and our identity over time. His arguments are complex and have wide ranging implications for practical ethics. However, at a number of points, Parfit made a further claim that is relevant for this paper:

‘This change of view also has psychological effects. It makes me care less about my own future, and the fact that I shall die. …I welcome these effects. Metaphysics can produce the consolations of philosophy’ (Parfit, 1984, p. 451).

The notion of philosophical ‘consolation’ is usually traced back to a work by the Roman statesman Boethius, who wrote ‘*De consolatione philosophiae*’ while imprisoned and awaiting trial (and eventual execution) (Boethius, 2009). Boethius’s work focused on questions of fate, good and evil, human nature and justice.

As Boethius imagined, and later writers have echoed, philosophy might be soothing for a variety of sources of unhappiness or anxiety. However, in relation to mortality it is worthwhile distinguishing two different claims:

### Rational Consolation

Philosophical analysis leads to the conclusion that it would be rational to care less about death than we currently do.

### Psychological Consolation

Philosophical reflection will actually lead us to care less about death than we currently do.

These claims may come apart. Obviously, it would be possible for philosophy to offer rational consolation, but to have little or no psychological traction. Equally, it is possible that a particular form of consolation might be psychologically effective, but have no rational basis (eg. supernatural explanations).

It is worth clarifying what exactly we are targeting with this consolation. Thomas Nagel captured the common intuitive feeling. Death is the permanent cessation of our existence. It is bad because ‘life is all one has, and the loss of it is the greatest loss one can sustain’ (Nagel, 1970, p. 73). Nagel argued that death is quintessentially a deprivation − it deprives us of valuable existence.^5^ The deprivation account of the badness of death accords with the widespread view that, for example, it is generally worse to die young.^6^

If death is bad in this way, how can philosophy offer consolation? Parfit appeared to believe that his view offered both rational and psychological consolation. This was on the basis of two separate strands of arguments.

## Self-lessness^7^

In the third part of Reasons and Persons, Parfit defended what he called the ‘Reductionist view’ about personal identity.^8^ On this account, whether a future person is ‘me’, and whether future experiences are mine (rather than someone else’s) is reducible to whether there is physical and psychological continuity between me now and that future person. There is no additional fact that determines identity, as would be the case if we believed in a Cartesian ‘ego’, or a soul. Importantly, this view has several implications that might be relevant for rational consolation.

Firstly, what matters to us prudentially is not all or nothing, rather it is a matter of degree. Future experiences might have stronger or weaker connections to my current self, and on Parfit’s account, I would have reason to have more concern for the former. As a consequence, I may come to care less about some of the future that death would prevent me from experiencing.^9^

Secondly, if we reflect on our life as a whole, it may be natural to think of it as a succession of selves rather than a single self (Parfit, 1984, pp. 305,319). For example, we might think of our child self, our young adult self, our self with young children, our middle-aged self, etc.

This is not a totally foreign idea. Literature and colloquial language sometimes refer to ‘an earlier self’ or ‘a later self’. However, if we come to think in that way, the end of our life may seem less singular, less profound. Our previous selves have already, in a sense, ceased to exist, while our current self will (if we continue to live) give way to other future versions.

‘Consider the fact that, in a few years, I shall be dead. This fact can seem depressing. But the reality is only this. After a certain time, none of the thoughts and experiences that occur will be directly causally related to this brain, or be connected in certain ways to these present experiences. That is all this fact involves. And, in that redescription, my death seems to disappear’ (Parfit, 1995, p. 45).

Finally, a diminishment of the strong sense of a single self, might simultaneously reduce the perceived difference between one’s own experiences and those of others. That is captured vividly in Parfit’s analogy of the dissolving walls of the glass tunnel. Towards the end of his life, Bertrand Russell (without necessarily sharing Parfit’s metaphysics) made a related claim: ‘The best way to overcome [a fear of death]… is to make your interests gradually wider and more impersonal, until bit by bit the walls of the ego recede, and your life becomes increasingly merged in the universal life’ (Russell, 1956, p. 52).

The significance, then, of Parfit’s revised understanding of identity is that it shifts us towards a more self-less attitude. This is rationally consoling since it diminishes the sense in which the life we have lived (and the future life that we might have experienced) is actually ours and blurs the boundary between oneself and others.

## Timelessness

In the second part of his book, Parfit analysed attitudes to time. This included the question of our attitudes to the near future versus the far future, but also a comparison of our attitudes to past and future experiences. The latter is particularly important for rational consolation because, ‘If we are afraid of death…the object of our dread is not our non-existence. It is only our future non-existence’ (Parfit, 1984, p. 175).

Parfit noted that we have deeply entrenched asymmetrical attitudes towards past and future pains and pleasures. We prefer our pains to be in our past and our pleasures to be in the future. We would often prefer (if we had a choice) to have a much greater pain in our past than a much smaller pain in our future. But as we move through life, more and more of our pleasures will lie behind us and fewer will be ahead. When we die, of course, there will be no further experiences of any kind to look forward to. Death deprives us of any future value. Moreover, we may well anticipate that the dying phase will include significant pains. Because of our asymmetrical view of past and future pains and pleasures, it is unsurprising that death looms as a frightening prospect.

However, it would be possible, and potentially beneficial, if we took a different approach. Parfit imagined someone, who he called ‘Timeless’, who regarded future and past experiences symmetrically. Timeless would be just as sad to learn that he had experienced pain in the past (if he had forgotten), as he would to discover that he was about to experience a similar pain in the near future. But importantly, Timeless would also lose his regret at loss of future as he approached his death. If we had such an attitude, ‘At any point in our lives we could enjoy looking either backward or forward to our whole lives’ (Parfit, 1984, p. 174).

In Reasons and Persons, Parfit was somewhat ambiguous about whether he thought that we *ought* to adopt a temporally neutral stance to our future and past wellbeing. He admitted that he, like everyone else, regarded his past suffering with relative indifference (Parfit, 1984, p. 173). However, in some places, he suggested that it would be better for us if we were to be temporally neutral. It would offer a consolation. Faced with the news that we would die tomorrow ‘We should not be greatly troubled…for though we now have nothing to look forward to, we have our whole lives to look back to’ (Parfit, 1984, p. 176). In later work, he made the stronger claim that ‘the most rational attitude is temporal neutrality’ (Parfit, 2011, pp. Volume 1, 495 fn 457).

If we were to take a more neutral attitude towards time this would undermine the sense in which death represents an ending, and diminish at least some of our concern about the loss of future value. Our death would not deprive us of our *past* experiences. In this way, timelessness would take the psychological sting out of mortality. We would still have reason to regret a premature death − since we would overall experience less wellbeing. However, adopting a timeless attitude would mean that if, for example, I learn that I am due to die from a genetic illness next month at age 49, I should no more fear that prospect than I would have had reason to fear it if I received the news a decade earlier, when I still had ten years to live.^10^

In combination, Parfit’s two insights yield a Rational Consolation for personal mortality.

### Parfitian rational consolation

Adopting a reductionist understanding of personal identity and a more timeless attitude, would mean that it is rational to care less about one’s own death or that of others.

Parfit claimed that it was at least possible for these to yield a psychological consolation:

‘Now that I have seen this, my death seems to me less bad.’ (Parfit, 1984, p. 281).

In Reasons and Persons, Parfit did not directly address whether the philosophical consolation might extend to sadness at the death of another. However, his letter to Joyce Oates suggests that he thought that it did. In that correspondence, Parfit indicated that he found it helpful to recall that the death of a loved friend did not diminish the reality of their (past) existence, any more than someone moving permanently overseas diminishes the reality of their (current) existence.^11^

If we adopt an approach that is like that of Timeless, separation from another person in time would not necessarily feel any different from physical separation. We could continue to enjoy the knowledge of our past shared experiences in the same way that we might (if they had not died) enjoy knowledge of future shared experiences. Furthermore, drawing on the reductionist view, we could redescribe the end of our loved one’s existence. There will continue to be some memories about their life, thoughts that are influenced by theirs, and actions and choices taken that result from their past actions. There will not be future experiences that are directly physically and psychologically connected to those experienced by the loved one in the past. But that, on Parfit’s account, is all that there is to the fact that they are no longer alive.

## Inconsolation

‘though Derek Parfit scarcely knew me, he wrote to me when my husband Raymond Smith died; a philosopher’s consolation which would seem to some people unfeeling, heartless & even tactless, but to the philosopher whose beliefs were his deepest self, logical’ - Joyce Carol Oates^12^

Does this sort of perspective actually console? One response might be to reject a key premise. Parfit’s views about identity and time have been and continue to be debated. Of course, if his arguments turn out not to be sound, then they cannot offer rational consolation. My aim, for this paper, is not to review all of the possible responses to Parfit nor to assess whether he is correct. Rather I will evaluate first whether, if he is correct, a Parfitian perspective provides reasons to care less about death (ie rational consolation). I will review in particular a couple of authors who have raised concerns that Parfit’s consolation might prove too much. That will turn out to be relevant to application of the consolation to grief. I will then turn to the plausibility of such a view offering psychological consolation.

## Rational inconsolation

Mark Johnston, in his book ‘Surviving Death’, criticised one of Parfit’s key arguments for reductionism about identity (Johnston, 2010, pp. 306-311). As noted, Parfit claimed that concerns about personal identity could be reduced to questions about physical and psychological continuity. Identity, itself, does not matter. However, Johnston argued that the value of a higher property can be more than the value of its constituents. There could be a further fact about personal identity, even if personal identity is constituted by physical and psychological continuity. As a form of reductio ad absurdum, Johnston suggested that Parfit’s argument would lead to ethical nihilism (Hummel, 2019). Since all physical facts depend on facts about atoms, particles and sub-atomic particles, but those fundamental particles do not themselves matter ethically − this appears to mean that nothing matters. If that were true, caring less about identity and mortality might come at the cost of caring less about everything.^13^

Parfit, in a response to Johnston, made a distinction between different types of constitutive relationships (Parfit, 2007). Conceptual relationships *can* mean that higher order descriptions are entirely constituted by lower order properties − in this case identity being conceptually constituted by physical and psychological continuity. In contrast, the relationship between higher order properties and fundamental particles is not conceptual. Consequently, there may be value in groupings and arrangements of particles (for example into a human person), even if the particles themselves lack such value. This enabled him to maintain the reductionist claim about identity, without being drawn to normative nihilism.

However, a different concern may arise from this response (Hummel, 2019, p. 78). Parfit’s consolation arises in part from his deflationary account of personal identity. Parfit redescribes death in terms of the lack of ongoing physical and psychological connections, and finds consolation in this. But Patrick Hummel has argued that this seems to undermine the claimed significance of the consolation. A mere conceptual redescription ‘seems to treat language as more important than reality’(Hummel, 2019, p. 78). Can it really offer any benefit to ourself or to others if philosophy’s consolation is merely semantic?

An opposite possibility is that the reductionist account of identity might give *increased* reason rather than decreased reason to fear death. Reduced connection to the future, and the idea of a succession of selves might mean that individuals face the prospect of ‘dying many times before their deaths’ (an attitude ascribed to cowards by Shakespeare’s Julius Caesar). Indeed this is one possible explanation of the surprising empirical evidence that I will summarise in the next section.

The above criticisms focus only on Parfit’s view of identity. But as outlined above, the other significant component is his view on our attitude towards time. It is the combination of these views that seems to give force to the Parfitian consolation. However, if we were to take a temporally neutral approach to past and future goods and bads, one concern is that this may radically undermine key elements of our psychology. For example, Samuel Scheffler has raised questions about the effect of Timelessness on our motivation. If we could enjoy looking back to pleasurable experiences in our past, why would we need to seek out such experiences in our future (Scheffler, 2021, p. 91)? He also expressed concern about the effect of such an attitude on personal relationships. ‘How would this affect people’s interactions and their desires to spend time together? How would it affect the structure of human attachments and our response to loss?’ (Scheffler, 2021, p. 91) Parfit’s response to the last of Scheffler’s questions is that the Timeless attitude might indeed diminish the pain of loss − this is one of the claimed benefits temporal neutrality. However, if (as Scheffler hints) this comes at the cost of failing to build, nurture and develop deep reciprocal relationships, that might not speak overall in favour of such an approach.

Scheffler admits that these concerns do not necessarily undermine the rationality of Parfit’s consolation (Scheffler, 2021, p. 94). He cites Parfit: ‘the rationality of an attitude does not depend on whether it is bad for us’ (Parfit, 1984, p. 177).^14^ However, Scheffler’s points seem particularly pertinent to the application of the consolation to grief. Parfit’s consolation reduces the apparent badness of death by reducing our emotional attachment to our own life, and reducing the strength of our desire for future existence. If that applies also to the death of loved ones, it seems that the consolation must come from reducing our emotional attachment to them and to reducing our desire that they exist in the future. But that might not be something that would overall be a good thing to aim for. This addresses directly one of Parfit’s arguments in favour of his consolation. He suggests that on any plausible moral view it would be better if we were all happier. But that would only speak in favour of a temporally neutral perspective if his prescribed changes in mindset were actually to lead to us all being happier. If, on the other hand, such a shift would reduce our wellbeing, we would have reason to avoid such a change.

## Psychological inconsolation

If a more self-less and timeless attitude were rational, that does not resolve whether it would actually provide solace − either in the face of personal mortality, or the death of others. Although philosophers have speculated on the plausibility of this claim,^15^ it is ultimately an empirical question.

## Self-less inconsolation

In fact, some recent empirical work appears directly relevant, at least to one element of Parfit’s consolation. Shaun Nichols and colleagues, in a study published in Cognitive Science, sought to examine the relationship between attitudes to personal identity and the self and attitudes towards death (Nichols et al., 2018). They had hypothesised that individuals who had a strong sense of the enduring self would be more fearful of death than those who had strong belief that the persisting self is an illusion. The study sought the views of more than 500 participants including lay volunteers from the US (mostly Christian or non-religious), orthodox Hindus and lay Buddhists from India and Bhutan, and Tibetan Buddhists from monasteries in India. The participants were first asked to indicate how strongly they felt that they were connected to a future version of themselves in a week, a year or 5 years’ time. As anticipated, there were significant differences between the groups. The American respondents felt strongly connected to their future selves, albeit with some reduction over long passages of time, while the monastic Tibetan Buddhists indicated a very low sense of connection. Hindu respondents, non-monastic Tibetans and Bhutanese respondents had views that were in-between. There were broadly similar responses when asked about belief in a ‘core self’ that persists over time; again monastic Tibetans indicated a view similar to Parfit’s reductionist account of personal identity. What is more, the Tibetan respondents (and virtually none of the American respondents) reported using this belief in the lack of a unitary self to help them in coping with the fear of death. It seemed that they were seeking consolation in line with Parfit’s claims. However, paradoxically (and to the surprise of the investigators), this did not translate into any actual reduction in their fear of death. Using standard scales for assessing the fear of personal death, the researchers found no difference overall in fear between the monastic Buddhists and the other religious and non-religious participants. They were just as likely to indicate fear of their future demise. Even more strikingly, the Buddhist monks had higher responses on questions relating to annihilation of the self (for example endorsing the statement that ‘Dying 1 year from now frightens me because of the loss and destruction of the self’), than any other group (Nichols et al., 2018).

What should we make of this response? On the face of it, it appears a striking refutation of Parfit’s psychological consolation. A group of those who have the strongest belief in the lack of persisting personal identity and who claim that they use this belief to assuage their fear of death actually report *higher* levels of death anxiety. This suggests that this particular form of philosophical consolation is impotent, possibly even counterproductive.

Like other pieces of empirical evidence, there are different potential interpretations. It might not be generalisable, and merits replication in other groups of Buddhists or non-Buddhists who ascribe to the no-self view of personal identity. Perhaps the no-self belief does not diminish fear, but reduces its significance or psychological traction?^16^ Alternatively, perhaps there are other features of monastic life that increase anxiety about mortality, counteracting the benefit of the attitude towards self. For example, perhaps regular meditation on the possibility of dying, increases its salience and engenders anxiety? However, even if not completely conclusive, this evidence does raise serious questions about the psychological consolation of self-lessness.

Let us assume, for the sake of argument, that the evidence described above is confirmed. What explanation might we give for failure of the Parfitian consolation?

One possibility is that the rational consolation itself is misdirected; it may misunderstand the source of our anxiety about death. As an example of this type of problem, Jeffrie Murphy, in an influential paper, noted that one popular form of philosophical consolation reminds us that once we are dead we will feel no pain. However, this consolation is ineffective (Murphy called it ‘inane’) since “it is quite obvious on reflection that fear of death and fear of pain are quite distinct” (Murphy, 1976, p. 196). Similarly, self-lessness might not provide psychological consolation if the loss of the future self is not the (principal) source of our fear of death. Why might this be the case? One reason is that our fear is not necessarily focussed on the loss of a distant future − the years of life that might accrue to psychologically disconnected selves. Instead, it may be that our fear of death is focussed much more proximally, on the loss of a future to which we are closely connected. If I come to accept the reductionist view, I may not be particularly concerned about the prospect of dying today and missing out on the experiences that I would have had in five years. But I may still be fearful (and possibly even more fearful) about the prospect of dying today, and missing out on the experience that I would have had tomorrow and next week. In this way, it may be that self-lessness offers psychological consolation for the abstract fear of mortality (the fact that I will die), but little for the tangible fear of dying soon. A different explanation might arise from a competing account of the badness of death. For example, Nagel’s view was that the badness of death was attributable to the loss of possibilities: “death, no matter how inevitable, is an abrupt cancellation of indefinitely extensive possible goods” (Nagel, 1970, p. 80). He further suggested that we should understand this in a relational way that is not confined to the boundaries of experience. Thus, the loss of future experience (even in the very far future) is bad for the previously living person.^17^ Alternative accounts of the badness of death (like Nagel’s) may not be compatible with a reductionist account of personal identity.

Finally, yet another possible explanation might come from the connection (or lack thereof) between rationality and fear. It may be that the Parfitian consolation targets the right reasons. It may indeed give us less reason to fear our death. However, that does not translate into any less anxiety. One claim (attributed particularly to psychologists and psychoanalysts) is that it is the nature of our psychology (and perhaps of our emotions) that rational understanding of death does not yield genuine comfort (Murphy, 1976, p. 188). We are very familiar with the idea that some fears (for example phobias) are extremely recalcitrant to reason (Brady, 2009). Perhaps the fear of death is of this kind.

## Timeless inconsolation

To my knowledge, there are no similar studies to the one described above of timeless attitudes and fear of death. There are some that support the idea that future-bias is widespread (at least in terms of personal pleasures and pains) (Greene et al., 2021). But no studies have evaluated whether attempts to reduce future bias would impact death anxiety. It is impossible, therefore, to empirically evaluate whether or not adopting a more timeless attitude would actually diminish fear of death. However, we might be sceptical. A number of philosophers have claimed that it would be impossible or inconceivable to renounce our temporal bias.^18^ Kaufman writes: “it might not be possible to become like Timeless and remain recognisably human” (Kaufman, 1999). At the very least, it seems extremely challenging to achieve consolation through a timeless attitude.

The fact that a belief (eg in timelessness) is difficult to achieve, does not mean that if it were achieved it would not be helpful. (It is even possible that a hard-won belief may have a greater psychological impact than a more-easily achievable belief).^19^ However, this difficulty may be relevant to our reflections in two ways. Firstly, it makes it hard to defend (or refute) the psychological consolation since there is little or no empirical evidence. Secondly, this difficulty may reduce the accessibility and *impact* of the claimed consolation. If timelessness is so difficult a mindset to reach, this may not, in practice, help many people.

I have concentrated on philosophical and psychological consolation in terms of personal mortality. I have not argued that philosophical insights into identity or time could not assuage fear of dying in some cases. Parfit claimed to have experienced such a benefit. Even if the empirical evidence cited is correct, it does not prove that consolation is impossible, just that perhaps it is unlikely.

However, if we now move our focus to distress at the loss of a loved one (particularly someone else’s distress at that loss), a stronger claim is warranted. It is, I suggest, deeply implausible that philosophical perspectives of the sort described in the first part of this paper could provide meaningful consolation.

## Grief and inconsolation

The Parfitian consolation provides reasons that potentially diminish fear of personal mortality. On the reductionist account, death is less bad, less of a threat to the self, because there is a less of a self to lose. Timeless would not feel dismayed as death approaches since even if there were fewer pleasures to look forward to, he could enjoy (in retrospect) more of the benefits in his past. However, fear of personal death and grief at death of another are not strictly parallel emotions. Grief is not purely or simply about the badness of death and loss of future value for the individual who has died. It is, after all, coherent and not uncommon to grieve deeply even after the death of someone for whom death is desired (for example, where an individual has a severe and untreatable illness). The intensity of our feelings of grief are not necessarily greater for unexpected or premature deaths than for those that occur at the end of a long life. Consider the nature of a widow’s grief for her partner at the end of a long marriage (Holm et al., 2019). These feelings are not closely or straightforwardly related to deprivation for the deceased and how bad death is for them (how much future life or wellbeing the individual who had died is deprived of).^20^ They are much more about how bad the death is for those left behind. Grief, fundamentally an emotion of loss and separation, is linked closely to the intensity and nature of the relationship with the deceased. To put it another way, grief is inextricably agent relative (Cholbi, 2017, p. 258).^21^ A philosophical consolation that is focused on the impartial badness of death is likely to have little traction on an emotion that is, itself, focused on something quite different.

Of course, the fact that grief is typically agent relative and focused on relationships does not mean that a philosophical consolation that aims to make the grieving person reflect in a more detached, self-less or time-neutral way would not be rational. Michael Cholbi has argued that grief is rational insofar as it is fitting to the object that is grieved, and the relationship with the deceased (Cholbi, 2017, Cholbi, 2022). If that is correct, one way that Parfit’s analysis might help is by reconceiving what it means for us to have relationships with another. On the reductionist account, what it means to be a father or a friend, a lover, a brother or a child is less distinctive, less singular. There is not a single ‘me’ who has a relationship with a single ‘them’. Rather there is a sequence of overlapping and connected ‘me’s that have interactions and connections over a period of time with another sequence of overlapping and connected ‘them’s.

But one potential problem with this consolation for grief is that it might, paradoxically, increase rather than decrease the sense of loss. The religious consolation soothes with the news that you have not lost your loved one for ever, they are still with you, you will see them again. In contrast, the Parfitian consolation implies almost the opposite − you never had the loved one, they were never with you, indeed there is not even any ‘you’ left behind. One worry is that the nature of this attempted consolation could exacerbate the sense of desolation, separation and loneliness of the bereaved.^22^

In a famous Buddhist work, ‘The life of Milarepa’, the 11^th^ century Tibetan yogi Milarepa describes his feelings on returning to his hometown and discovering the remains of his mother: ‘When I realized they were the bones of my mother, I was so overcome with grief that I could hardly stand it. I could not think, I could not speak, and an overwhelming sense of longing and sadness swept over me’ (Gtsang-smyon et al., 2010).

There is another story about the 18^th^ century Buddhist disciple Satsujo:

‘When Satsujo, a great disciple of Hakuin, was old, she lost her granddaughter, which grieved her very much. An old man from the neighborhood came and admonished her: ‘Why are you wailing so much? If people hear this, they’ll all say, ‘the old lady …was enlightened, so now why is she mourning her granddaughter so much?’ … Satsujo glared at her neighbor and scolded him: ‘You baldheaded fool, what do you know? My tears and weeping are better for my granddaughter than incense, flower, and lamps!” (Fischer, 2013)

Such anecdotes obviously do not show that the Buddhist doctrine of ‘non-self’ (or its secular philosophical equivalent) cannot console in the face of grief. But perhaps they speak to the difficulty of this − even for those like Milarepa and Satsujo who have studied the doctrines for many years.^23^

But perhaps a timeless attitude would be more useful in the face of grief? Parfit drew the analogy between physical and temporal separation. We could imagine our loved ones as just being temporally absent − akin to being in another room or another place.^24^ Moreover, such a change might offer something like the religious consolation. Our friend of twenty years is not gone. They still exist − just not in the present. We can see them again − in our past. Indeed, if we were fully able to embrace timelessness, we could in theory appreciate those twenty five years in our shared past just as much as if we now were able to look forward to 25 years with them in a shared future.^25^

This seems like it might console. However, one real challenge of this form of consolation is its accessibility. Our asymmetrical attitudes towards time are deeply ingrained. If it were possible to modify them, that might require a great deal of intense effort, time and practice.^26^ Perhaps the most that could be achieved is some reduction in the asymmetrical nature of our attitudes to past and future.^27^ Yet if this were attained, it would then at most diminish and not remove our grief at the loss of future with a loved one.

Moreover, even a temporally neutral mindset (and perhaps here I reveal my own temporal bias), would fail to genuinely assuage some of the things that the bereaved find most difficult, most painful. Even if they could enjoy remembering their past shared times, there would be no new ones. The survivor cannot feel the embrace or touch of their loved one, cannot (except through recordings) hear their voice. They cannot share feelings or seek the deceased’s advice. For those who have been lost at a young age, the survivor will never see them grow up, develop their own tastes and loves and hates, never share with them heart break and disappointment, excitement and achievement. One source of sadness for at least some bereaved is the sense of unfinished business, things that needed to be spoken about or done with the deceased. But by their nature, such regrets cannot be assuaged by backward reflection on those things that were actually said and done.

I have suggested that the pain of grief typically arises from the loss of a relationship. Niko Kolodny has argued that there are three defining features of relationships that form the basis of love: such relationships obtain between particular people over time, they are historical (they depend upon facts about shared pasts), and they are ongoing (Kolodny, 2003).^28^ It is particularly this last element that links to the pain of grief. Death causes a disconnection with the loved one. There can be no ongoing reciprocal or mutual relationship with the person who has died.^29^ Indeed, that is one reason for thinking that Parfit’s analogy with spatial dislocation is misplaced. Separation from loved ones by great distance can itself be associated with considerable emotional distress (Miller et al., 2018). It may not be much consolation to imagine that the deceased is in New Zealand.

Further, it is not simply that the deceased is temporally displaced in a foreign country. They are distant, and each day moving away from us − as if they were passengers on the Pioneer space probe, propelled by momentum ever further from Earth and forever out of radio contact. For the survivor, past memories of the dead person may still be enjoyed, but those memories gradually and inexorably fade. Then the bereaved may experience renewed sadness as they struggle vainly to hold on to the threads of the shared past.

Kolodny’s account of relationships, and much of what I have described above about the pain of grieving is ineluctably temporally rooted. On the one hand, this might seem to reinforce the potential power of Parfit’s consolation. If we could shake off our temporal shackles, we might thereby become inured to loss. However, the opposite conclusion seems (to me) more plausible. Timelessness is highly unlikely to succeed because much of the special value of love and the special pain of grief is so closely connected to way that we form reciprocal connections with other people over specific periods of time.

The Parfitian consolation is neither simple, nor easy. That is one reason why, when it comes to fear of our own death or sadness at the loss of another, that this consolation may not succeed. But that difficulty applies even more if consolation were to be offered to another person, someone who was not a philosopher and had not read Reasons and Persons. Joyce Carol Oates recognised the genuine sentiment behind Parfit’s note to her. But she also described its potential insensitivity. Such a negative impression is by no means unique to philosophical consolation. Many of those who have experienced loss describe the wide range of ways that others can act insensitively even if well-intentioned (Pogue, 2019). For example, friends, co-workers, or family might try to diminish or underplay the perceived loss (‘it could have been worse’), offer unhelpful exhortations (‘you need to move on’), or express hollow-sounding expressions of fratitude (‘I know how you feel’). But there is a particularly jarring form of consolation that is sometimes offered where the other person attempts to offer solace on the basis of religious beliefs that the bereaved does not share. To suggest to the mourning atheist that they should not be sad because their lost child ‘is in a better place’, or ‘this is all part of God’s plan’, is not only unhelpful, it can be deeply disrespectful and distressing. This sort of problem potentially also applies to some non-religious beliefs. It might apply to the sort of philosophical consolation that is the focus of this paper.^30^ Simply put − it might be consoling for the bereaved to discuss identity and temporal neutrality if they hold certain compatible pre-existing metaphysical beliefs. However, if they hold those beliefs they are already (potentially) consoled by them.^31^ If they do not share those beliefs, for a third party to offer them is likely to be at the very least ineffective, and at the worst offensive.

## Conclusions

I have sought in this paper to examine the nature and effectiveness of a particular form of philosophical consolation in the face of death of the self or of others. Philosophy for the most part, if it tries to offer consolation, does not deny that we die; rather it seeks to provide reasons why mortality is not as bad as feared. One way of doing this − as exemplified in Parfit’s approach, is to move us to a more impartial and impersonal perspective. If we are able to move outside our individual perspective, or recognise that there is no unitary self, death may be less of a threat. However, there is some evidence to suggest that such a shift does not necessarily translate into less death anxiety. Moreover, applied to grief at loss of another, such a perspective may be misdirected. Grief is deeply linked to our relationships with loved others. As such, the impartial shift might only provide consolation at the cost of reduced attachment to those we love.

In contrast, a temporally neutral perspective, offers a theoretically powerful way of reducing the sense of loss at being temporally separated from a loved one, or the anxiety as death approaches. However, it is unclear whether it is psychologically possible to achieve such a perspective. Even if it were possible, it may not diminish the pain of separation − particularly as memories fade. Finally, there is a powerful sense that sophisticated philosophical insights cannot offer acute relief to those who do not already share them. If we wish to console our grieving friend or family member, we may need something other than metaphysics.

I have suggested that Parfit’s philosophical insights about the self and time may not console − particularly in terms of the grief of another. That may seem a somewhat dispiriting conclusion. I close with some tentative suggestions about a much more modest form of consolation.

Reflection on why a self-less and timeless shift does not console − helps us to recognise the deeply personal and temporal nature of grief. If we wish to support those who are grieving, we should recognise and acknowledge the value of the relationships that they are mourning and their real pain at their loss. We can, as far as we are able, and as far as they wish us to, accompany them in the present moments of their grieving and help them to share and relive their memories of the lost loved one. We can accept and remain supportive when grieving takes time.

Philosophy can do more than detach us from sources of suffering and sadness. It can also motivate and direct us in ways to care for those who are suffering and sad. Our consolation can then be informed by philosophy − rather than being strictly philosophical. We may not be able to offer our grieving companion rational consolation, but we can try to rationally console.

## Footnotes

1 Letter to Joyce Carol Oates. Quote from correspondence in epigraph, (Oates, 2012)

2 One exception are the *consolatio* letters written by Stoic philosophers to grieving friends or family members, for example those by Seneca and Plutarch.

3 Some transhumanist philosophers point to the possibility of surviving somatic demise eg through mind- uploading or cryogenics. In this paper, I focus on consolation in the absence of such technologies.

4 As I note below, there are important overlaps between Parfit’s consolation and that offered in Buddhist philosophy. That may suggest that this analysis is also relevant for some forms of religious consolation.

5 Parfit supported the deprivation view − ‘Some think that [non-existence] itself is bad. But the more plausible view is that its only fault is what is causes us to lose’ (Parfit, 1984, p. 176).

6 See, for example (Solberg et al., 2016) (McMahan, 1988).

7 In this paper, I use the term “self-lessness” as shorthand for Parfit’s Reductionist theory of identity, and to contrast it with the “timeless” elements of this work. As noted, this theory reduces the sense of a single self. (This is distinct from the altruistic sense in which the word ‘selflessness’ is commonly used.’

8 In defence of this view, Parfit worked through a series of thought experiments and arguments that lie beyond the scope of this paper.

9 The impartial badness of death is not changed by the Reductionist identity account. It would be just as bad for someone to die prematurely at age 30 and miss out on 50 years of good life, whether or not we accept Parfit’s view. However, on Parfit’s account, it would be much less bad *for the 30 year old*.

10 Although Parfit believed that a timeless attitude would diminish our anxiety as death approaches, it is conceivable that it might have the opposite effect earlier in life. For example, I might become just as anxious or distressed to learn that I will die in 10 years, as I currently would be if I were to learn that I have only a month to live.

11 see footnote 1. Parfit’s analogy with spatial separation might be a claim about rational consolation − we have no more reason to care about temporal than physical distance from our loved ones. However, in his letters, he appears to be making a more explicitly psychological claim. As a result of his rational consolation he found it helpful to consider death as similar to separation in space, and found this eased his sadness. I return to the plausibility of this claim below.

12 Oates, JC. Jan 3 2017. Twitter. https://twitter.com/JoyceCarolOates/status/816333589864476677?s=20&t=op0PbovTddvrunnb2oYEew

13 Interestingly, although Johnston disagreed with Parfit on a key argument, he ultimately shares with Parfit a view that there are no persisting selves (Johnston, 2010, p. 306). Furthermore, he notes that this view is likely to offer some consolation. For people who are ‘less attached to their own individual personalities…the obliteration of their own individual personalities is less of a loss… In this sense, a reasonably good person is more able to face down or overcome death’ (ibid. 318).

14 The fact that an attitude is bad for us does not mean it is irrational, and vice versa. However, the effect of an attitude on our wider life (including our overall wellbeing) might give us reasons to try to adopt it, or not to. Elsewhere in Reasons and Persons, Parfit accepted that ‘The fact that an attitude is bad for us … [can] show that we should try to change this attitude.’ (Parfit, 1984, p. 179).

15 Parfit admitted that his revisionary attitudes were hard to believe, though thought that it was possible. Caspar Hare has made the stronger claim that ‘you may as well try to lose your skin’ as try to rid yourself of the bias towards the future (Hare, 2013).

16 In a separate part of the study, the authors examined respondents willingness to forego a life-preserving treatment for the sake of a stranger. The monastic Tibetans were (contrary to expectation) least likely to do this. This appears to imply that the increased fear of death is associated with more self-preserving intuitions.

17 Nagel gave the example of a man who develops a severe brain injury leaving him with the capacities of an infant (Nagel, 1970, p. 77). Although in this new state, the man is weakly (if at all) psychologically connected to his previous self, this injury seems particularly bad for the (pre-impaired) person who thereby misses out on a range of possibilities that are no longer available to him.

18 (Hare, 2013) (Rosenbaum, 1989). (Scheffler, 2021).

19 I am grateful to an anonymous reviewer for suggesting this

20 As another example, grief after stillbirth late in pregnancy appears to be much more profound than grief after miscarriage (Cuisinier et al., 1993).

21 Nussbaum writes: ‘Internal to the grief itself must be the perception of the beloved object and of her importance; the grief itself must estimate the richness of the love between us, its centrality in my life.’ (Nussbaum, 2003, p. 44)

22 One possibility, that I do not have space to explore further here is the relationship between grieving and identity. Nussbaum notes that one of the exquisite challenges of recovery from grief is the threat to identity. ‘as one reweaves the fabric of one’s life after a loss, and the as the thoughts around which one has defined one’s aims and aspirations change tense, one becomes to that extent, a different person’ (Nussbaum, 2003, p. 83) One possibility is that the reductionist account of identity might exacerbate rather than ease such concerns, and even make recovery from grief more difficult, since on such an account (compared with a non-reductionist account) it is more conceivable that recovery from grief would yield a different future self. The bereaved would have reason to mourn the loss of their pre-bereavement self.

23 Nichols et al speculate that the strong innate sense of continuity within a biological life may be resistant to the philosophical insight that there is not an enduring self (Nichols et al., 2018, p. 330).

24 Those who are bereaved sometimes describe a sense that the deceased are merely absent − out at work, or on a trip or elsewhere. But this is not typically described as a consolation, nor is it meant literally. Rather, it reflects a disorienting sense of unreality as well as difficulty in coming to terms with the finality of their loss.

25 It obviously, would not be exactly the same, since if we were to have another 25 years with our friend, that would give us a total of 50 years together.

26 There is a further concern that the crucible of grief is particularly inhospitable territory for philosophical reflection. Michael Cholbi makes the point that those in the throes of grief may find it particularly hard to reflect in the detached way that philosophy might require (Cholbi, 2022, p. 14).

27 One reason that a completely symmetrical timeless attitude is impossible, is that (as pointed out by Scheffler), motivation, intention and large parts of normativity are necessarily based on forward projecting thoughts and the (future) consequences of our actions. Since our actions can only affect the present and future, (and not the past) our concerns are necessarily going to be strongly future oriented. Caspar Hare has argued that such a bias may be a brute fact of our psychology (Hare, 2013).

28 Kolodny’s account is focused on those relationships that provide the basis for love − eg friends, lovers, family members. He does not discuss grief, but there is strong overlap between an analysis of the basis for love and the basis for grief (Parkes, 2013). (One common observation is that ‘grief is the price that we pay for love’). It is possible that grief might arise in the absence of love. However, the conclusions that I draw will apply to the majority of cases.

29 Millar and Lopez-Cantero have argued recently that relationships can continue following bereavement (Millar et al., 2022) − though they must take a different form. Love shifts to being unreciprocated, and relationships change to being one-sided. If this is correct, the pain of grieving is not the complete loss of a relationship − rather the loss of a reciprocal relationship.

30 In his reflections about the value of philosophy for personal fear of death, Mark Johnston has argued that such insights should not be extrapolated to mourning: “It would be ham-fisted and obscene to try to interfere with the logic of mourning by prematurely appealing to the irrationality [of a particular way of thinking of the deceased]” (Johnston, 2010, p. 50).

31 In reference to a different form of philosophical consolation, Cicero made a similar remark: ‘I pass over the method of Cleanthes, since that is directed at the wise person who does not need consoling. For if you managed to persuade the bereaved person that nothing is bad but shameful conduct, then you have taken away not his grief, but his unwisdom. And this is not the right moment for such a lesson’ (Cicero et al., 2002, p. 77).
